# Control of the Phase Distribution in TMDs by Strain Engineering and Kirigami Techniques

**DOI:** 10.1021/acs.jpclett.4c03464

**Published:** 2025-01-15

**Authors:** Arun Jangir, Duc Tam Ho, Udo Schwingenschlögl

**Affiliations:** † Physical Science and Engineering Division, King Abdullah University of Science and Technology (KAUST), Thuwal 23955-6900, Saudi Arabia; ‡ Department of Mechanical and Construction Engineering, Northumbria University, Newcastle Upon Tyne NE1 8ST, United Kingdom

## Abstract

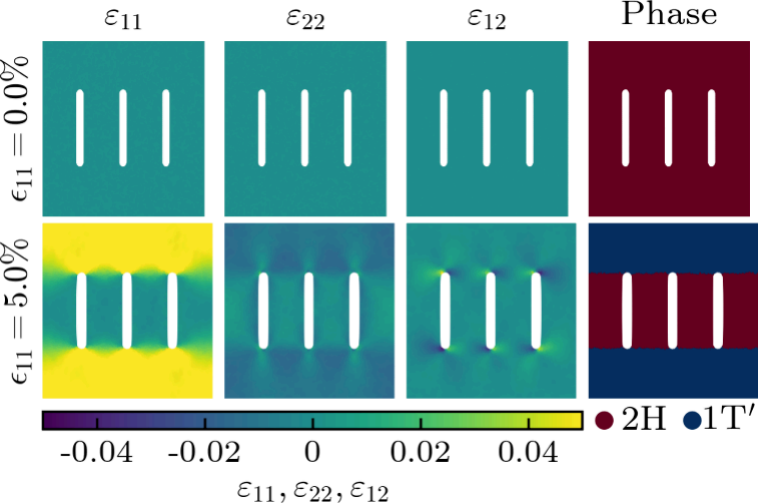

Materials exhibiting both metallic and semiconducting states, including two-dimensional transition metal dichalcogenides (TMDs), have numerous applications. We therefore investigate the effects of axial and shear strains on the phase energetics of pristine and striped TMDs using density functional theory and classical molecular dynamics simulations. We demonstrate that control of the phase distribution can be achieved by the integration of strain engineering and Kirigami techniques. Our results extend the understanding of the phase energetics in TMDs and reveal an effective strategy for creating virtually defect-free metal-semiconductor-metal junctions.

Two-dimensional transition metal dichalcogenides (TMDs) consist of a layer of metal atoms sandwiched between layers of chalcogen atoms. Depending on the specific arrangements of the metal and chalcogen atoms, different phases exist, such as the trigonal prismatic (2H) phase, octahedral (1T) phase, and distorted octahedral (1T′) phase.^1^ The 2H phase is energetically favorable for group VI and VII TMDs, while the 1T phase is energetically favorable for group IV and X TMDs.^2^ TMDs can exhibit phenomena such as superconductivity,^3^ the quantum spin Hall effect,^4^ ferromagnetism,^5^ thermoelectricity,^6^ charge density waves,^7^ ferroelectricity,^8^ valley polarization,^9^ quasi-metallicity,^10^ and piezoelectricity.^11^ Their scope of application, therefore, is enormous, spanning fields such as oscillators,^12^ gas sensors,^13^ logic circuits,^14^ photodetectors,^15^ catalysts,^16^ supercapacitors,^16^ batteries,^16^ and memristors.^17^ Application in logic circuits specifically requires metal-semiconductor-metal junctions with high-quality ohmic contacts. Metastable phases of TMDs can be accessed by strategies such as strain engineering,^18−20^ electrostatic gating,^21^ electron injection,^22^ chemical treatment,^23^ and direct growth.^24^ Control over the phase distribution in a sample is possible by femtosecond laser treatment^25^ and precursor-guided synthesis.^26^

While prior studies on strain engineering have focused on the effects of homogeneous axial strain,^18−20^ more complex strain configurations, such as shear strain, may have significant implications for the phase stability and phase transitions. Inhomogeneous strain additionally has the potential to spatially control the phase distribution for creating metal-semiconductor-metal junctions. Inspired by the Japanese art of cutting paper, inhomogeneous strain can be generated by a combination of strain engineering and Kirigami techniques, as there exist experimental approaches to cut two-dimensional materials with a precision of up to a few nanometers.^27^ While so far only phase transitions in pristine and Janus TMDs have been studied,^18,28^ TMDs can also form striped phases. For these reasons, this study combines density functional theory with classical molecular dynamics simulations to predict the phase energetics and analyze the phase transitions of pristine and striped TMDs under axial and shear strains, both homogeneous and inhomogeneous.

Density functional theory calculations are conducted using the projector augmented wave method of the Vienna ab initio simulation package,^29^ adopting the Perdew–Burke–Ernzerhof (PBE) exchange correlation functional. The converged plane wave cutoff and Monkhorst–Pack k-grid turn out to be 600 eV and 9 × 15 × 1, respectively. Monkhorst–Pack k-grids of similar density are chosen to conduct phonon calculations for 3 × 5 × 1 supercells by the Phonopy package.^30^ The convergence threshold of the total energy is set to 10^–7^ eV. All structures are optimized by the variable cell method until no atomic force component exceeds 10^–3^ eV/Å. Using the same termination criterion, the structure optimizations are continued by the fixed cell method applying a Green-Lagrangian strain tensor , where **F** is the deformation tensor and **I** is the identity matrix.^31^**F** = **C**_*s*_ · **C**_*u*_^–1^, where **C**_*s*_ and **C**_*u*_, respectively, are matrices consisting of the cell vectors of the strained and unstrained unit cells (as columns). When the unstrained unit cell is orthorhombic with the in-plane lattice parameters *a*_*u*_ and *b*_*u*_ and the strained unit cell is monoclinic with the in-plane lattice parameters *a*_*s*_ and *b*_*s*_ and the monoclinic angle γ_*s*_, we have
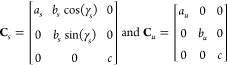
1where *c* is the out-of-plane lattice parameter, and the corresponding strain components are
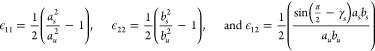
2We study ϵ_11_ and ϵ_22_ values from −0.10 to 0.10 in steps of 0.02 and ϵ_12_ values from 0.00 to 0.05 in steps of 0.01. The total energies of the optimized structures are recalculated using the Heyd-Scuseria-Ernzerhof (HSE) hybrid functional^32^ to evaluate the resilience of the computational methodology.

We employ the large-scale atomic/molecular massively parallel simulator package^33^ for conducting classical molecular dynamics simulations for the 2H phase of pristine MoTe_2_, using the Stillinger-Weber force field^34^ with the parameters given in ref (35). 90 × 156 × 1 simulation cells (approximately 50 × 50 nm) with periodic boundary conditions in the *x*- and *y*-directions are built for different cutting patterns by removing selected atoms. After equilibration with a Nosé–Hoover thermostat at 4 K and 0 atm for 50 ps, they are subjected to strain along the *x*-direction (ϵ_11_) with a rate of 10^9^ s^–1^. The spatial distribution of the atomic strain **ε** is obtained by the Green-Lagrangian method as implemented in the OVITO package^36^ (atomic displacements averaged within 12 Å around each atom) and used together with the total energy results of the density functional theory calculations to identify the phase in the different regions of the simulation cell. To this aim, bivariate spline and linear interpolations are used to obtain the total energies for intermediate values of the axial and shear strains, respectively.

The crystal structures of the 2H and 1T′ phases of the pristine and striped TMDs are depicted in Figure 1. The chalcogen atoms are arranged alternately along the *x*-direction (armchair direction) in the striped TMDs. Similar to ReS_2*x*_Se_2(1–*x*)_,^37^ it turns out that the 1T′ phase of the striped TMDs can realize two structural configurations labeled as 1T′-v1 and 1T′-v2 (Figure 1 (d)). The relaxed lattice parameters of the unstrained unit cell, total energy difference *ΔE* = *E*_1T′_ – *E*_2H_, band gap *E*_gap_, and nature of the band gap are presented in Table 1 for the 2H and 1T′ phases of pristine MoTe_2_ and striped MoSSe, MoSTe, MoSeTe, WSSe, WSTe, and WSeTe. The corresponding phonon band structures in Figure 2 show dynamic stability in each case. We find that striped MoSSe, MoSeTe, and WSSe favor the 2H phase following pristine MoS_2_, MoSe_2_, MoTe_2_, WS_2_, and WSe_2_, while striped WSTe and WSeTe favor the 1T′ phase following pristine WTe_2_.^18^ Striped MoSTe favors the 1T′ phase because of the availability of two crystallographic sites to accommodate the significant size disparity between the chalcogen atoms. According to the electronic band structures in Figure 3, all 2H phases are semiconductors, with band gaps ranging from 1.08 to 1.61 eV, while the 1T′ phases are metals, except for semiconductors with very small band gaps.

**Figure 1:**
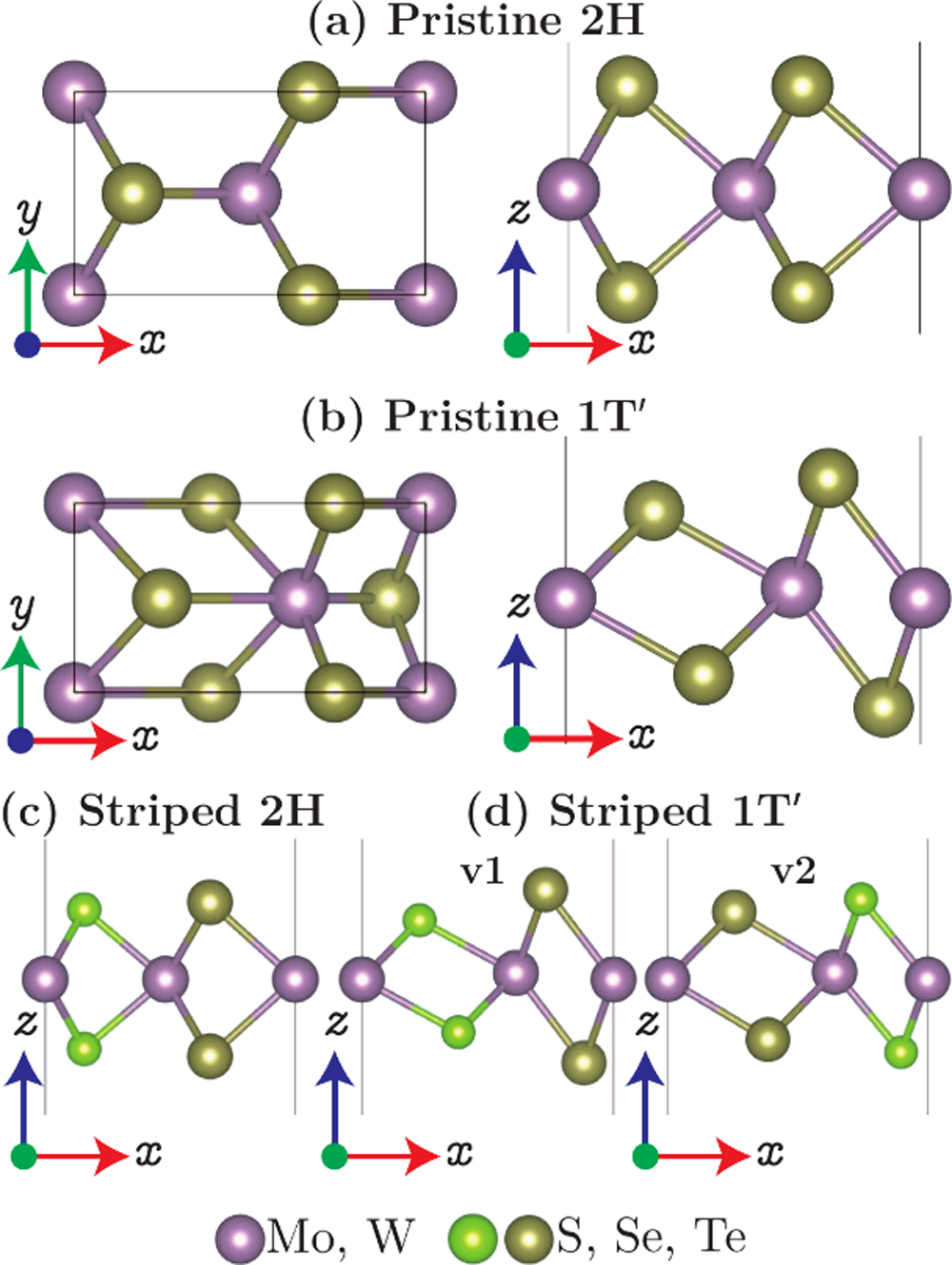
Crystal structures of the (a,c) 2H and (b,d) 1T′ phases of the pristine and striped TMDs.

**Table 1:** Lattice Parameters *a*_*u*_ and *b*_*u*_, Total Energy Difference *ΔE* = *E*_1T′_ – *E*_2H_, Band Gap *E*_gap_, and Nature of the Band Gap for the 2H and 1T′ Phases of Pristine MoTe_2_ and Striped MoSSe, MoSTe, MoSeTe, WSSe, WSTe, and WSeTe (for Configurations v1 and v2)

Material	Phase	*a*_*u*_ (Å)	*b*_*u*_ (Å)	PBE *ΔE* (meV)	PBE *E*_gap_ (eV)	HSE *ΔE* (meV)	HSE *E*_gap_ (eV)	Nature
Pristine MoTe_2_	2H	6.15	3.55	0	1.08	0	1.51	Direct
	1T′	6.37	3.45	84	-	53	-	Metallic
Striped MoSSe	2H	5.62	3.25	0	1.51	0	1.98	Direct
	1T′-v1	5.77	3.23	997	0.07	1121	0.12	Indirect
	1T′-v2	5.93	3.21	704	-	771	-	Metallic
Striped MoSTe	2H	5.78	3.37	0	1.17	0	1.59	Indirect
	1T′-v1	5.90	3.27	795	-	892	-	Metallic
	1T′-v2	6.30	3.20	–17	-	–17	-	Metallic
Striped MoSeTe	2H	5.93	3.44	0	1.16	0	1.60	Indirect
	1T′-v1	6.06	3.37	573	-	610	-	Metallic
	1T′-v2	6.32	3.31	49	-	36	-	Metallic
Striped WSSe	2H	5.62	3.25	0	1.61	0	2.10	Direct
	1T′-v1	5.76	3.24	902	0.02	1064	0.11	Indirect
	1T′-v2	5.91	3.23	668	-	766	-	Metallic
Striped WSTe	2H	5.79	3.37	0	1.21	0	1.64	Indirect
	1T′-v1	5.83	3.32	633	0.04	727	0.12	Direct
	1T′-v2	6.27	3.23	–66	-	–57	-	Metallic
Striped WSeTe	2H	5.94	3.44	0	1.19	0	1.61	Indirect
	1T′-v1	6.02	3.39	359	0.05	405	0.10	Indirect
	1T′-v2	6.27	3.35	–103	-	–112	-	Metallic

**Figure 2:**
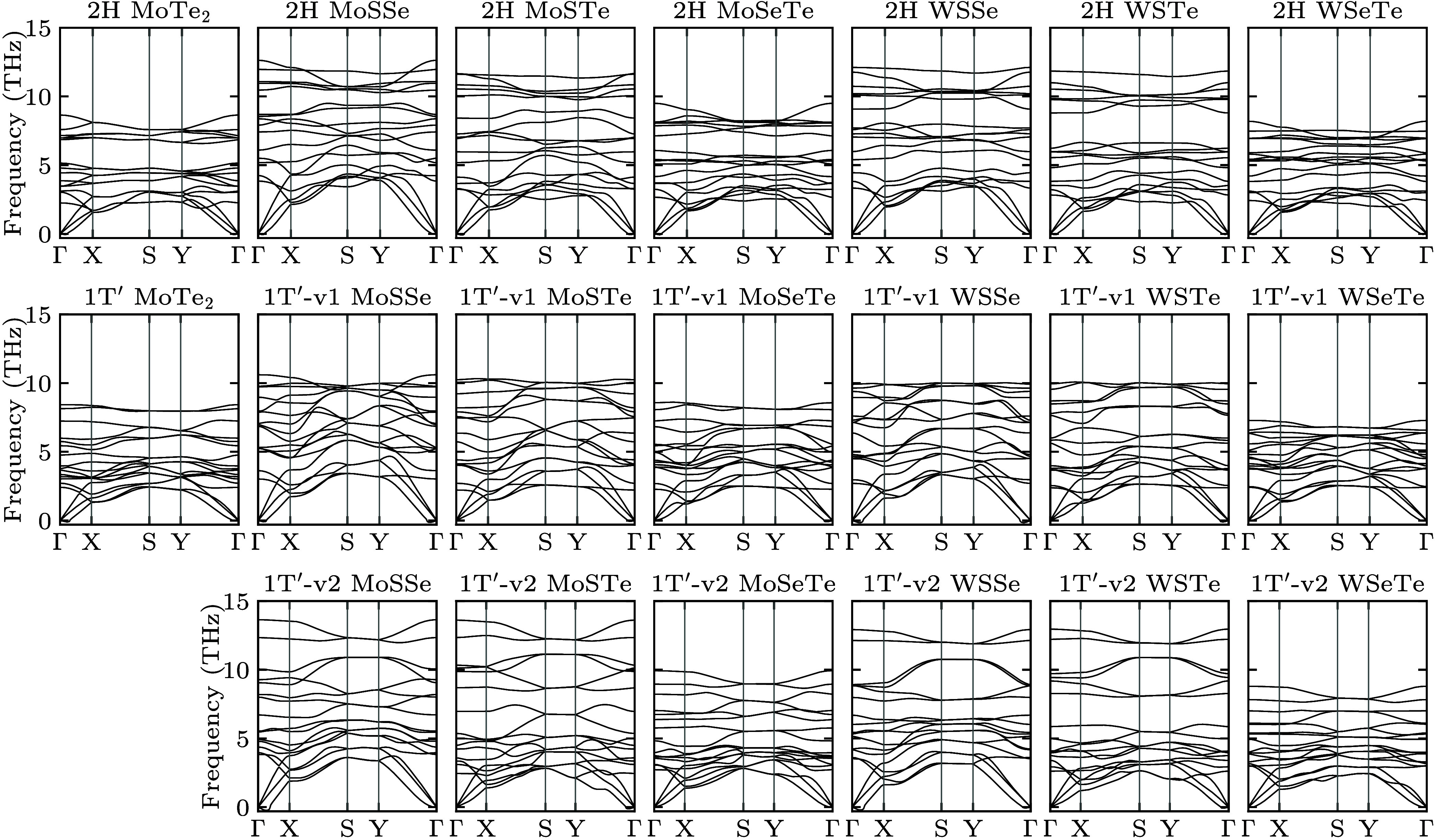
Phonon band structures along the high symmetry path Γ→X→S→Y→Γ of the unstrained 2H and 1T′ phases of pristine MoTe_2_ and striped MoSSe, MoSTe, MoSeTe, WSSe, WSTe, and WSeTe (for configurations v1 and v2).

**Figure 3:**
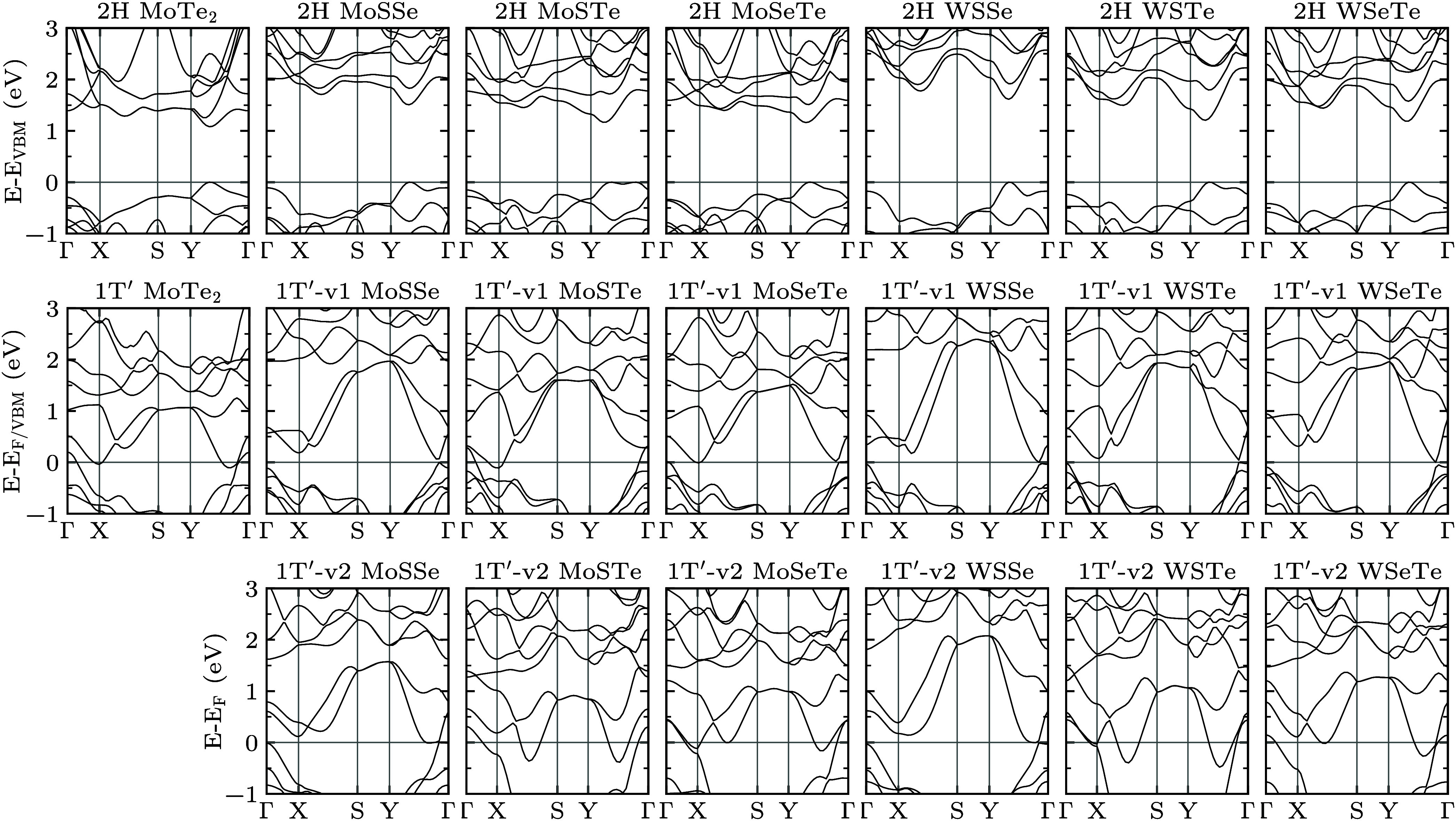
Electronic band structures along the high symmetry path Γ→X→S→Y→Γ of the unstrained 2H and 1T′ phases of pristine MoTe_2_ and striped MoSSe, MoSTe, MoSeTe, WSSe, WSTe, and WSeTe (for configurations v1 and v2).

According to Figure 4, the 2H phase of pristine MoTe_2_ is energetically favorable over the 1T′ phase by 84 meV. The 1T′-v1 phase exhibits higher *ΔE* than the 1T′-v2 phase for all the striped TMDs, which is consistent with the experimental observations for ReS_2*x*_Se_2(1–*x*)_.^37^ As the 1T′ phase of pristine MoTe_2_ and 1T′-v2 phases of striped MoSTe, MoSeTe, WSTe, and WSeTe exhibit low *ΔE*, these materials are chosen for studying phase transitions. The effects of strain on *ΔE* are addressed in Figure 5 for both the PBE and HSE functionals. Distinct differences between the two functionals are observed only for MoTe_2_, because in this case *ΔE* is 31 meV higher for the PBE than for the HSE functional while in the other cases this difference is much smaller (Table 1). We find for the PBE functional that a transition of pristine MoTe_2_ from the 2H into the 1T′ phase can occur at an axial strain of less than 5% in the absence of shear strain, while the presence of shear strain increases the required strain. The same behavior is observed for the HSE functional, but a lower axial strain is sufficient to trigger the phase transition. A transition from the 2H into the 1T′ phase can also occur for all of the striped TMDs at an axial strain of less than 5%. The PBE functional again consistently predicts higher axial strain to trigger the phase transition than the HSE functional. Interestingly, higher axial strain is required for striped MoSTe and MoSeTe to induce the phase transition than for striped WSTe and WSeTe despite their lower |*ΔE*| (see Figure 4), likely due to the significantly negative *ΔE* of WSTe and WSeTe, which makes the 1T′ phase of these materials profoundly favorable over the 2H phase. Shear strain has a similar effect in the case of the striped TMDs as in the case of pristine MoTe_2_.

**Figure 4:**
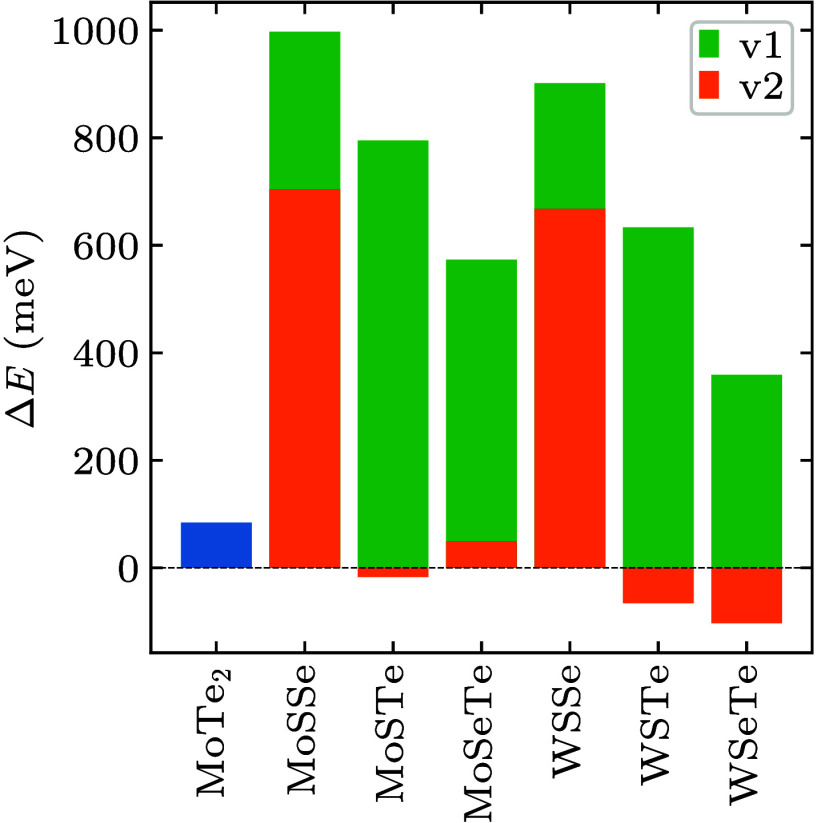
Total energy difference *ΔE* = *E*_1T′_ – *E*_2H_ of pristine MoTe_2_ and striped MoSSe, MoSTe, MoSeTe, WSSe, WSTe, and WSeTe (for configurations v1 and v2).

**Figure 5:**
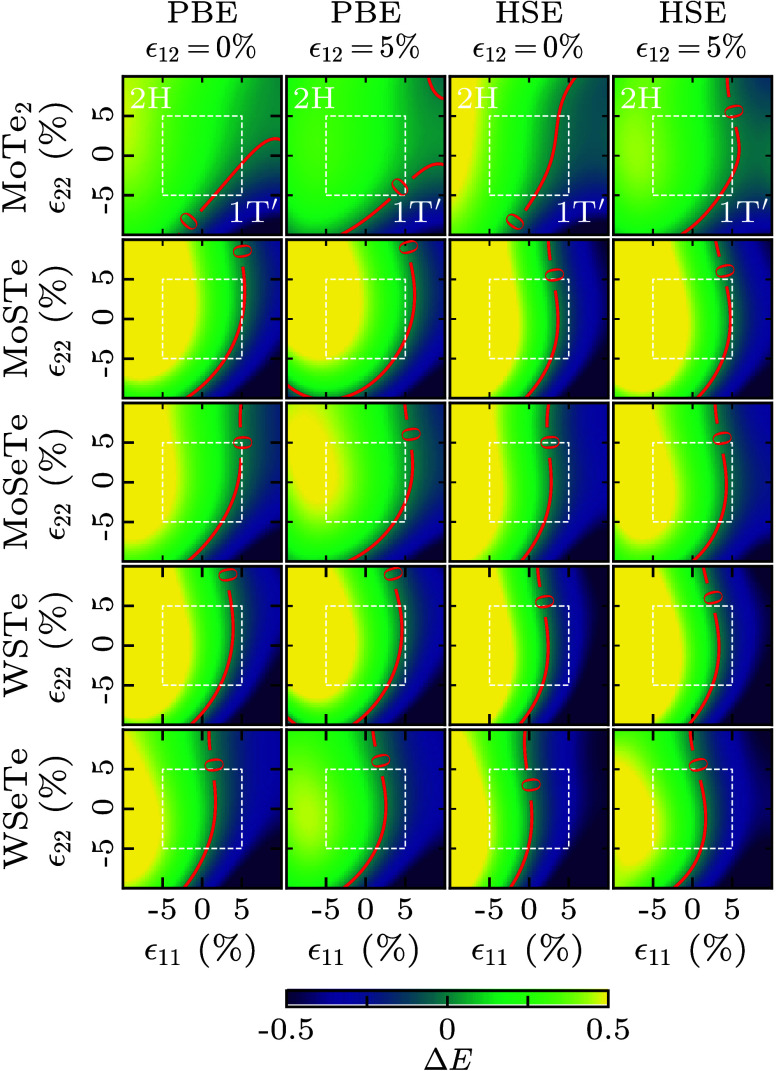
Total energy difference *ΔE* = *E*_1T′_ – *E*_2H_ (in eV) of pristine MoTe_2_ and striped MoSTe, MoSeTe, WSTe, and WSeTe (for configuration v2) under different shear strains (ϵ_12_) as obtained for the PBE and HSE functionals. The white dotted square marks the 5% axial strain region. The zero isoline is shown in red.

Through molecular dynamics simulations, we next explore the impact of various cutting patterns on the phase behavior of pristine MoTe_2_ under axial strain (ϵ_11_). The obtained atomic strain distributions and corresponding phases identified using the total energy results of the density functional theory calculations are presented in Figures 6 and 7 for straight and Bézier cutting patterns, respectively. Results for triangular and circular cutting patterns are shown in Figures S1, S2, and S3. The cutting patterns are found to create pronounced strain gradients, which determine the distributions of the 2H and 1T′ phases. In the case of straight cutting, small islands of the 1T′ phase appear at 2.5% axial strain close to the ends of the cuts (where ε_11_ is higher, i.e., the 1T′ phase is energetically more favorable). They grow until at 5% axial strain a distribution emerges with the 2H phase remaining between the cuts and the 1T′ phase consolidated outside of the cutting pattern. The phase boundaries are virtually perfect, representing defect-free metal-semiconductor-metal junctions. Further increase of the axial strain beyond 7.5% results in the development of cracks, which are clearly seen in the atomic strain distribution at 10% axial strain. The Bézier cutting pattern also induces coexistence of the 2H and 1T′ phases, but the phase boundaries are more complex. The resistance against cracks, on the other hand, is enhanced to above 10% axial strain. These results highlight that the design of the cutting pattern is highly critical to balance between phase separation and mechanical robustness. Different cutting patterns also lead to different edges with their respective electronic properties (metallic for zigzag and semiconducting for armchair^38^). Overall, the integration of strain engineering and Kirigami techniques emerges as an effective strategy for the simultaneous optimization of the phase distribution and mechanical robustness of pristine and striped TMDs.

**Figure 6:**
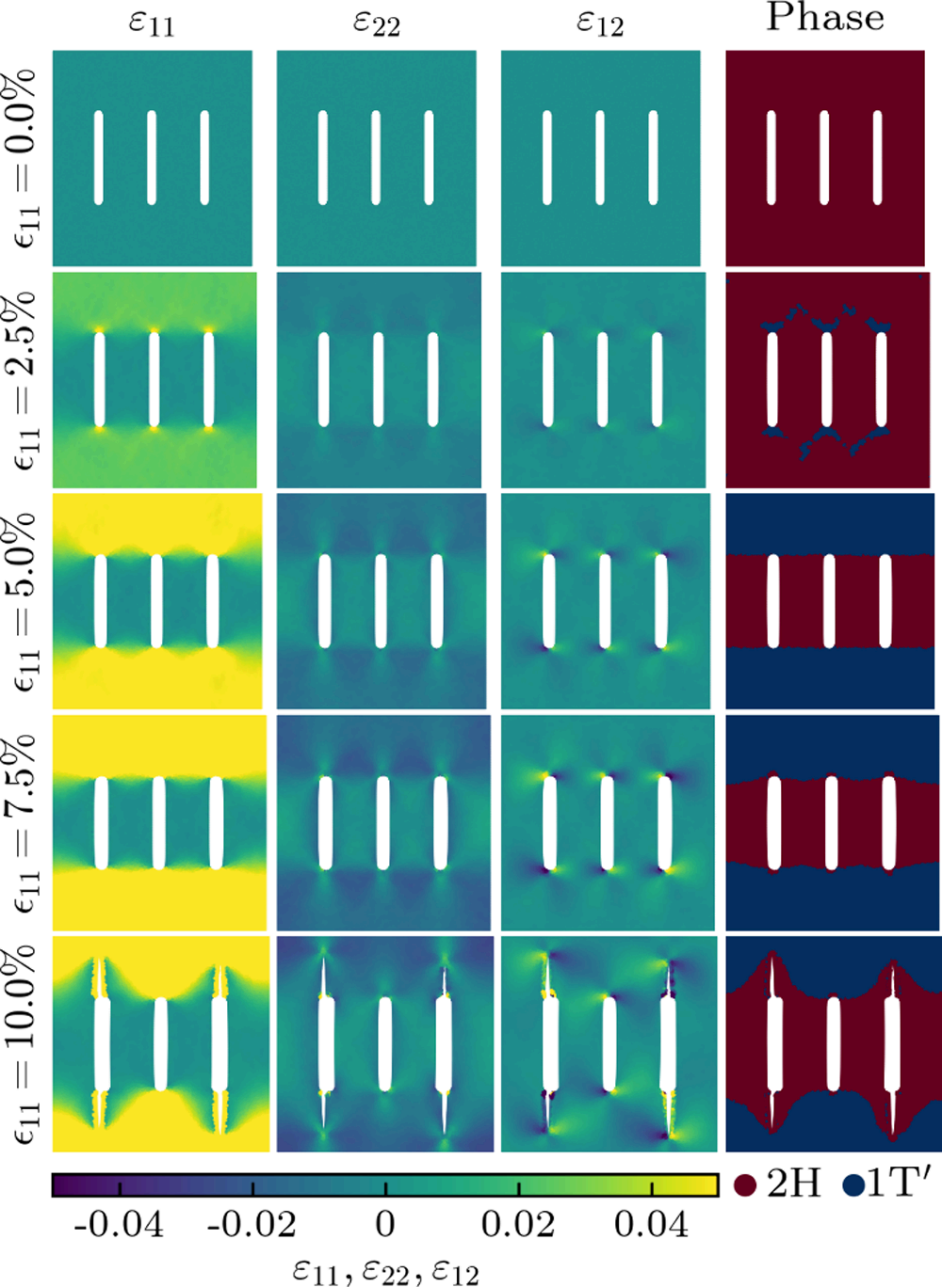
Atomic strain distributions and corresponding phases in pristine MoTe_2_ under different axial strains (ϵ_11_) in the presence of straight cutting. The white areas are regions without atoms.

**Figure 7:**
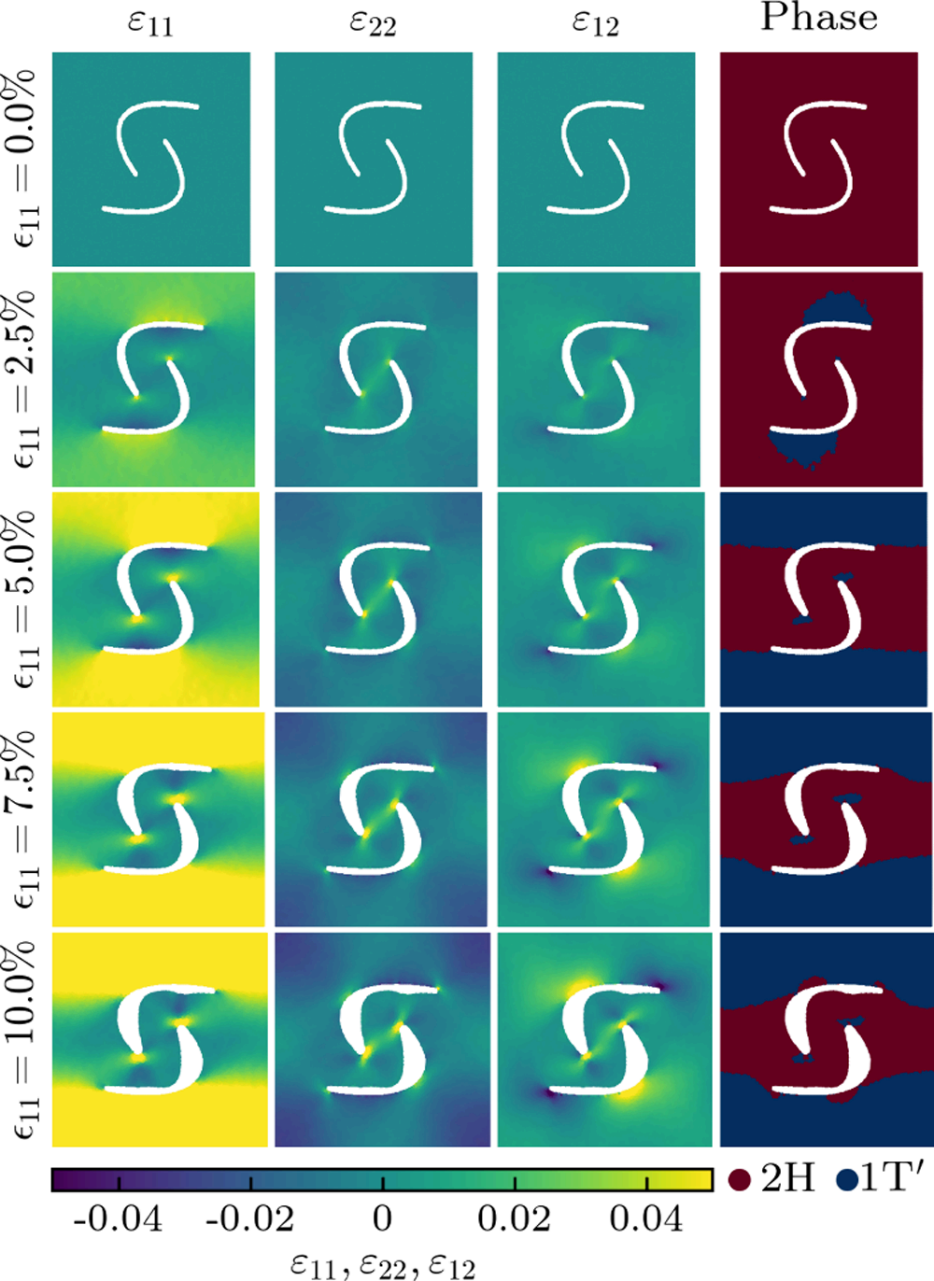
Atomic strain distributions and corresponding phases in pristine MoTe_2_ under different axial strains (ϵ_11_) in the presence of Bézier cutting. The white areas are regions without atoms.

In conclusion, we have comprehensively analyzed the phase energetics and phase transitions in TMDs under axial and shear strains (homogeneous and inhomogeneous) for pristine MoTe_2_ and various striped TMDs. It turns out that a phase transition from the 2H into the 1T′ or 1T′-v2 phase can be consistently achieved by less than 5% axial strain. Shear strain necessitates a higher axial strain to trigger the phase transition. Molecular dynamics simulations show a significant impact of the cutting pattern. If mindfully designed, cutting patterns therefore can be used to control the phase distribution and mechanical robustness. Importantly, virtually defect-free metal-semiconductor-metal junctions can be formed by this approach, giving rise to a new toolkit for the development of materials for electronic devices.

## Supporting Information Available

Results for triangular and circular cutting patterns (PDF)
