# Supplementary material for: Control of the Phase Distribution in TMDs by Strain Engineering and Kirigami Techniques
Source: J Phys Chem Lett. 2025 Jan 15;16(3):811–7. doi: 10.1021/acs.jpclett.4c03464 (PMC11770754; doi:10.1021/acs.jpclett.4c03464)
Supplement: Supplementary file 1 — jz4c03464_si_001.pdf [file jz4c03464_si_001.pdf]

Supporting Information
Control of the Phase Distribution in TMDs by Strain Engineering and Kirigami Techniques

Arun Jangir[1], Duc Tam Ho[2], and Udo Schwingenschlögl[1,*]

[1]*Physical Science and Engineering Division,*

King Abdullah University of Science and Technology (KAUST),

Thuwal 23955-6900, Saudi Arabia and

[2]*Department of Mechanical and Construction Engineering,*

Northumbria University, Newcastle Upon Tyne NE1 8ST, United Kingdom

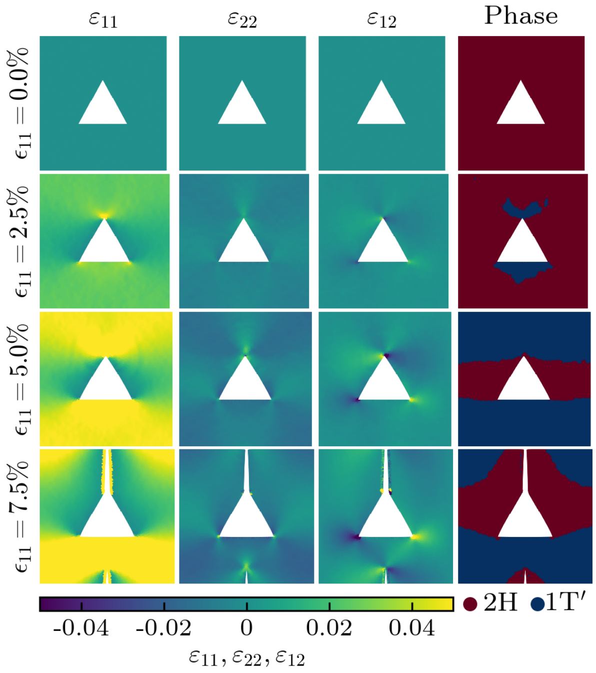

FIG. S1. Atomic strain distributions and corresponding phases in pristine $MoTe_2$ under different axial strains (ϵ_{11}) in the presence of triangular cutting. The white areas are regions without atoms.

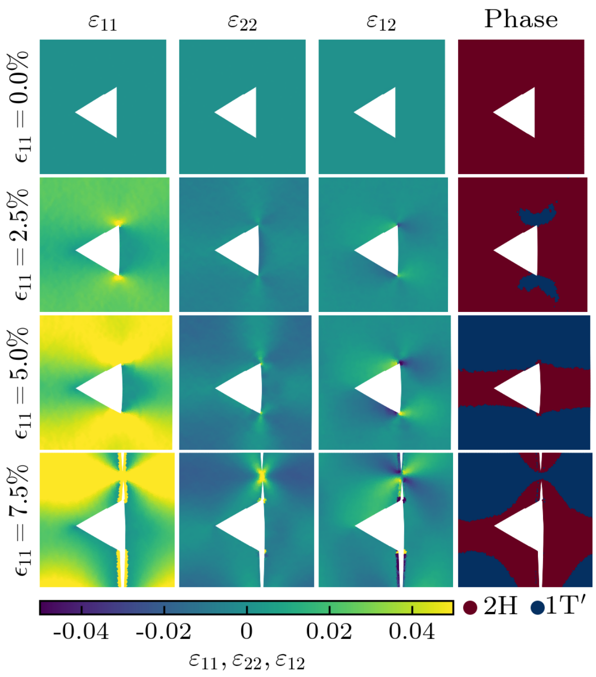

FIG. S2. Atomic strain distributions and corresponding phases in pristine $MoTe_2$ under different axial strains (ϵ_{11}) in the presence of triangular cutting. The white areas are regions without atoms.

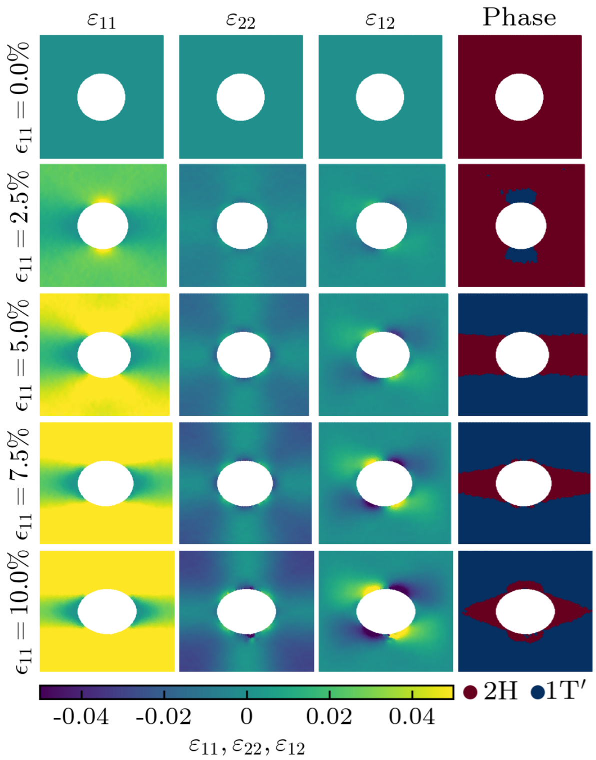

FIG. S3. Atomic strain distributions and corresponding phases in pristine $MoTe_2$ under different axial strains (ϵ_{11}) in the presence of circular cutting. The white areas are regions without atoms.
